# Predictive power of splenic thickness for post-hepatectomy liver failure in HBV-associated hepatocellular carcinoma patients

**DOI:** 10.1186/s12957-017-1281-6

**Published:** 2017-12-04

**Authors:** Xiang Chen, Heng Zou, Li Xiong, Sheng-Fu Huang, Xiong-Ying Miao, Yu Wen

**Affiliations:** Department of General Surgery, The Second Xiangya Hospital, Central South University, Renmin Road 139, Changsha, 410011 Hunan People’s Republic of China

**Keywords:** Splenic thickness, Post-hepatectomy liver failure, Hepatitis B virus, Hepatocellular carcinoma, Mortality, Morbidity

## Abstract

**Background:**

The purpose of this case series is to investigate the relationship between splenic thickness (ST) and postoperative outcomes after hepatic resection in hepatitis B virus (HBV)-associated hepatocellular carcinoma (HCC) patients.

**Methods:**

The clinical data of 320 patients with HBV-associated HCC who had undergone liver resection were retrospectively analyzed. The value of ST in predicting postoperative outcomes was evaluated.

**Results:**

A total of 320 patients were enrolled in the study. An increase in ST was significantly associated with an increase in portal vein diameter (PVD), indocyanine green retention rate 15 min (ICG R15), and total bilirubin (TBIL); however, it was negatively correlated with platelet count (PLT). Post-hepatectomy liver failure (PHLF) occurred in 35 (10.9%) patients. Multivariate logistic regression analysis showed that ST was an independent predictor of morbidity and mortality after hepatectomy. Meanwhile, ST was associated with an almost sixfold increased risk for developing perioperative complications (OR 5.678; 95% CI 2.873 to 11.224; *P* < 0.001) and almost 13-fold increased risk for mortality after hepatectomy (OR 13.007; 95% CI 1.238 to 136.627; *P* = 0.033).The area under the receiver operating characteristic (ROC) curve (AUC) of ST for predicting the incidence of PHLF was 0.754 (95% confidence interval (CI) 0.667 to 0.841; *P* < 0.001), with a sensitivity of 57.1% and a specificity of 82.5%, which were significantly greater than those of the ICG R15 level (AUC 0.670; 95% CI 0.560 to 0.779; *P* < 0.001). The critical value of ST was 43.5 mm.

**Conclusions:**

ST, which is an easy, inexpensive, and routinely available perioperative marker, showed a favorable predictive value for postoperative outcomes in HBV-associated HCC patients.

## Background

Hepatocellular carcinoma (HCC) is the second leading cause of cancer mortality worldwide and the sixth most common type of cancer [1]. Liver resection is an accepted first-line curative treatment and is performed in most HCC patients. Although recent advances in diagnosis, surgical techniques, and perioperative treatments have positively improved the safety and outcomes of hepatic surgery [2], post-hepatectomy liver failure (PHLF) still occasionally occurs in clinical practice and remains the major cause of hepatectomy-related mortality [3]. The risk for PHLF is high in patients with chronic liver disease or cirrhosis, especially in those patients who have portal hypertension or thrombocytopenia [4, 5]. Hence, accurately assessing the safety of surgery prior to hepatectomy in these patients is imperative.

The prevalence of histologic cirrhosis is high among patients with hepatitis B virus (HBV) and hepatitis C virus (HCV) infections [6]. In Asia, approximately 80% of HCC cases occur in patients with cirrhosis derived from chronic HBV infection [7].These co-morbidities, along with portal hypertension [8], are associated with increased morbidity or mortality because of a significant impairment in liver function [9]. In addition, a palpable or enlarged spleen is common in these patients and is considered as an independent predictor of the presence of large esophageal varices, which carry a high risk of bleeding and allow the clinical progression of cirrhosis to be monitored [10]. Moreover, the liver-to-spleen ratio has been used to evaluate the severity of liver disease and predict post-hepatectomy complications [11]. Therefore, there is a need for preoperative spleen assessment during hepatic resection in HBV-associated HCC patients. This has resulted in the adoption of spleen size as a marker of the safety and prognosis of liver surgery [12–14].

However, using spleen size in the context of hepatectomy in HBV-associated HCC patients has not yet been investigated. Therefore, the present study assessed the use of the splenic thickness (ST) compared with the indocyanine green retention rate 15 min (ICG R15) level to predict PHLF and other postoperative outcomes in HBV-associated HCC patients undergoing hepatic resection.

## Methods

### Patients

Between November 2013 and January 2017, 366 patients underwent curative hepatic resection for HCC at the Second Xiangya Hospital of Central South University. The inclusion criteria for this study were as follows: good liver functional reserve with Child-Pugh (CP) grade A or B, no treatment for HCC before liver resection, positivity of hepatitis B surface antigen (HBsAg), and no cardiopulmonary, renal, or cerebral dysfunction before liver resection. The exclusion criteria were as follows: co-infection with HCV and/or human immunodeficiency virus, previous splenectomy, and any other known cause of splenomegaly (i.e., hematopathy, infections, inflammatory, or primary splenic diseases). Finally, a total of 320 patients were included in this study. The patients were stratified into the normal spleen group (ST < 40 mm) or the thickened spleen group (ST ≥ 40 mm).

All patients gave their informed consent to participate in the clinical study, and approvals were obtained from the ethics committee at Central South University.

### Perioperative management

Routine preoperative investigations were performed on all patients and included a thorough history, physical examination, routine blood tests, chest X-ray, ultrasonography, and contrast-enhanced computed tomography (CT) or magnetic resonance imaging (MRI) of the abdomen. A preoperative ICG clearance test and CT volumetry were undertaken routinely to evaluate the functional reserve of the future liver remnant. Anti-HBV treatment was administered to the patients following surgery as needed. Postoperative laboratory tests included routine blood tests, biochemical tests, and coagulation function tests and were performed on the first, third, fifth, and seventh days after surgery or thereafter as needed. Ultrasonography was performed on the fifth day after the surgery to detect whether there was pleural fluid or abdominal ascites.

### Definitions

Splenic thickness was measured by ultrasound and defined as the transversal distance between the porta lienis and the point of tangency of the lateral border, which was examined by a senior radiologist who had extensive experience in sonography. Two or three measurements were averaged when the thickness of the anterior and posterior portions of the spleen differed considerably. All patients were imaged while resting in a supine position with an empty stomach. The ultrasonographic measurement of splenic thickness was technically feasible in all patients.

Liver resection was classified as minor hepatectomy (segmentectomy and non-anatomic wedge resection of two segments or fewer) and major hepatectomy (three Couinaud segments or more) [15]. Liver cirrhosis and HCC were diagnosed by the pathological examination of the resected specimen. Clinically significant portal hypertension (CSPH) was defined by the presence of esophageal/gastric varices, splenomegaly (diameter greater than 12 cm on ultrasonography), or in patients with a low platelet count (PLT) (< 100 × 10^9^/l) [16]. The presence of esophageal varices (EV) was demonstrated by upper digestive endoscopy on all patients. PHLF was defined as a total serum bilirubin value > 50 μmol/l and a prothrombin time index < 50% (equal to an international normalized ratio (INR) > 1.7) on postoperative day 5 or thereafter, based on the International Study Group of Liver Surgery (ISGLS) classification [17, 18]. The model for end-stage liver disease (MELD) score was calculated using the following formula: 11.2 × ln (international normalized ratio) + 9.57 × ln (creatinine, mg/dl) + 3.78 × ln (bilirubin, mg/dl) + 6.43 × (etiology—0 if cholestatic or alcoholic, 1 otherwise) [19]. Postoperative complications were classified according to the Dindo-Clavien classification during the 30 days after hepatectomy [20].A major complication was defined as grade 3 or above and a grade 2 or less qualified as a minor complication. Mortality was defined as death occurring during the 30 days after liver resection.

### Statistical methods

Statistical analyses were performed using SPSS 17.0 (SPSS, Inc., Chicago, IL, USA). Categorical variables were presented as relative frequencies and percentages and were compared using the *χ*2 test or Fisher’s exact test. Continuous variables were expressed as the means ± standard deviation (SD) and were compared using the Mann-Whitney *U* test or Student’s test as appropriate. A statistically significant result was defined as *P* < 0.05, and the *P* values were two-sided. Univariate analysis and multivariate logistic regression analysis were performed to identify independent predictors for the development of PHLF and postoperative morbidity and mortality, and the adjusted odds ratio (OR) per standard deviation change and the 95% confidence interval (CI) were calculated. The predictive ability of ST and ICG R15 was assessed by the receiver operating characteristic (ROC) curve and the corresponding area under the ROC curve (AUC). The correlations between ST and other variables were tested by Spearman’s rank correlation coefficients.

## Results

### Characteristics of the patients

Demographic and clinical characteristics of 320 patients were presented in Table 1. A total of 285 (89.1%) men and 35 (10.9%) women had a median age of 51 years (range 17–77), and the median splenic thickness value of the entire study cohort was 37.5 mm (range 19–72). Among these 320 patients, 228 patients (71.2%) were diagnosed with liver cirrhosis after hepatectomy. According to the CP grade, the majority of patients had grade A (289/320, 90.3%), and the remaining patients had grade B. Based on the Dindo-Clavien classification, 68 (21.2%) patients developed complications, and 43 (13.4%) patients and 25 (7.8%) patients were classified as having minor and major complications, respectively.

**Table 1:** Characteristics of patients with normal or thickened spleen

Variables	No. of patients (*n* = 320)	Normal (*n* = 223)	Thickening (*n* = 97)	*P*
Age, years	51.4 ± 10.6	51.3 ± 10.9	51.7 ± 9.7	0.754
Gender				0.531
Male	285 (89.1%)	197 (88.3%)	88 (90.7%)	
Female	35 (10.9%)	26 (11.7%)	9 (9.3%)	
BMI, Kg/m^2^	22.6 ± 3.2	22.5 ± 3.1	22.8 ± 3.3	0.436
PLT, × 10^9^/l	156.7 ± 79.7	177.9 ± 76.1	107.7 ± 65.3	< 0.001
ALT, U/l	42.8 ± 32.8	41.2 ± 26.7	46.5 ± 43.6	0.267
AST, U/l	45.5 ± 24.2	44.9 ± 21.8	46.6 ± 28.9	0.575
ALB, g/l	38.2 ± 4.2	38.3 ± 4.0	38.1 ± 4.8	0.596
PT, s	13.1 ± 1.2	12.9 ± 1.2	13.5 ± 1.3	< 0.001
INR	1.1 ± 0.1	1.0 ± 0.1	1.1 ± 0.1	< 0.001
CREA, umol/l	73.9 ± 12.1	74.1 ± 12.1	73.8 ± 12.1	0.744
TBIL, umol/l	15.1 ± 7.2	13.9 ± 6.3	17.7 ± 8.5	< 0.001
Tumor size, cm	6.2 ± 3.2	6.4 ± 3.2	5.8 ± 3.2	0.131
Operation time, min	188.7 ± 59.8	187.8 ± 58.9	190.8 ± 62.1	0.684
ICG R15, %	7.4 ± 4.4	6.7 ± 3.3	9.2 ± 6.0	< 0.001
ST, mm	37.5 ± 9.2	32.4 ± 3.0	49.3 ± 7.7	< 0.001
Blood loss, ml	593.2 ± 702.1	563.7 ± 721.5	661.0 ± 653.9	0.255
PVD, mm	11.0 ± 1.9	10.5 ± 1.5	12.3 ± 2.3	< 0.001
PVT				0.958
Positive	50 (15.6%)	35 (15.7%)	15 (15.5%)	
Negative	270 (84.4%)	188 (84.3%)	82 (84.5%)	
EV				< 0.001
Positive	40 (12.5%)	12 (5.4%)	28 (28.9%)	
Negative	280 (87.5%)	211 (94.6%)	69 (71.1%)	
CSPH				< 0.001
Positive	88 (27.5%)	23 (10.3%)	65 (67.0%)	
Negative	232 (72.5%)	200 (89.7%)	32 (33.0%)	
Cirrhosis				< 0.001
Positive	228 (71.2%)	140 (62.8%)	88 (90.7%)	
Negative	92 (28.8%)	83 (37.2%)	9 (9.3%)	
Liver resection				0.126
Minor	261 (81.6%)	177 (79.4%)	84 (86.6%)	
Major	59 (18.4%)	46 (20.6%)	13 (13.4%)	
Complication				< 0.001
None	252 (78.8%)	203 (91.0%)	49 (50.5%)	
Minor	43 (13.4%)	11 (4.9%)	32 (33.0%)	
Major	25 (7.8%)	9 (4.0%)	16 (16.5%)	
Child-Pugh grade				0.021
A	289 (90.3%)	207 (92.8%)	82 (84.5%)	
B	31 (9.7%)	16 (7.2%)	15 (15.5%)	
MELD				
< 7.5	278 (86.9%)	203 (91.0%)	75 (77.3.0%)	0.001
≥ 7.5	42 (13.1%)	20 (9.0%)	22 (22.7%)	

*Abbreviation*: *BMI* body mass index, *PLT* platelet counts, *ALT* alanine transaminase, *AST* aspartate aminotransferase, *ALB* albumin, *PT* prothrombin time, *INR* international normalized ratio, *CREA* creatinine, *TBIL* total bilirubin, *ST* splenic thickness, *PVD* portal vein diameter, *PVT* portal vein thrombus, *EV* esophageal varices, *CSPH* clinically significant portal hypertension, *ICG R15* indocyanine green retention rate 15 min, *MELD* the model for end-stage liver disease

Data are expressed as means ± SD or *n* (%)

### Patient characteristics and operative details between the normal spleen group and thickened spleen group

In our cohort, 97 (30.3%) patients had a thickened spleen (ST ≥ 40 mm) (shown in Table 1), with a median ST value of 49.3 mm. Additionally, these patients had a significantly low preoperative PLT, long prothrombin time (PT), and increased INR. Patients in the thickened spleen group also had a significantly increased presence rate of cirrhosis (90.7 vs. 62.8%, *P* < 0.001) and esophageal varices (28.9 vs. 5.4%, *P* < 0.001), accompanied by a greater proportion of CP grade B (15.5 vs. 7.2%, *P* = 0.021) and higher MELD scores (22.7 vs. 9.0%, *P* = 0.001), compared with the normal spleen group. In this context, although fewer patients in the thickened spleen group experienced a major liver resection, they developed more major and minor complications than did the normal group.

### Patient characteristics according to the development of PHLF

A comparison of patient demographics and clinical variables in the PHLF group and non-PHLF group was presented in Table 2. PHLF occurred in 35 patients (10.9%). A lower PLT and greater total bilirubin (TBIL) were associated with patients who developed PHLF. A thickened spleen was significantly associated with the development of PHLF. Moreover, the underlying impaired liver that was associated with the cirrhosis significantly affected the development of PHLF, which was consistent with the increased ICG R15 in the PHLF group. In addition, more patients who developed PHLF had CSPH than those without PHLF (54.3% (19/35) vs. 24.2% (69/285), *P* < 0.001). Regarding the surgical factors, blood loss and the proportion of major liver resection were significantly greater in patients with PHLF, while the operation time was similar between the two groups.

**Table 2:** Study population and clinicopathological characteristics according to the development of PHLF

Variables	PHLF (−) (*n* = 285)	PHLF (+) (*n* = 35)	*P*
Age, years	51.2 ± 10.5	53.3 ± 11.1	0.255
BMI, Kg/m2	22.6 ± 3.1	22.3 ± 3.4	0.589
PLT, × 10^9^/l	161.7 ± 80.2	115.6 ± 63.0	0.001
ALT, U/l	41.9 ± 31.0	50.3 ± 44.2	0.153
AST, U/l	44.2 ± 22.8	55.5 ± 31.8	0.050
ALB, g/l	38.3 ± 4.2	37.2 ± 4.8	0.129
PT, s	13.1 ± 1.2	13.1 ± 1.3	0.946
INR	1.1 ± 0.1	1.0 ± 0.1	0.513
CREA, umol/l	74.6 ± 11.9	69.0 ± 12.9	0.010
TBIL, umol/l	14.6 ± 6.6	19.0 ± 10.7	0.020
Tumor size, cm	6.1 ± 3.2	7.3 ± 3.4	0.030
Operation time, min	187.5 ± 59.3	198.5 ± 63.8	0.307
ICG R15, %	7.1 ± 4.2	9.7 ± 5.4	0.010
ST, mm	36.5 ± 8.4	45.5 ± 10.9	< 0.001
Blood loss, ml	526.6 ± 502.5	1135.7 ± 1475.1	0.021
PVD, mm	11.0 ± 2.0	11.1 ± 1.4	0.800
PVT			0.223
Positive	47 (16.5%)	3 (8.6%)	
Negative	238 (83.5%)	32 (91.4%)	
EV			0.281
Positive	38 (13.3%)	2 (5.7%)	
Negative	247 (86.7%)	33 (94.3%)	
CSPH			< 0.001
Positive	69 (24.2%)	19 (54.3%)	
Negative	216 (75.8%)	16 (45.7%)	
Cirrhosis			0.108
Positive	199 (69.8%)	29 (82.9%)	
Negative	86 (30.2%)	6 (17.1%)	
Liver resection			0.036
Minor	237 (83.2%)	24 (68.6%)	
Major	48 (16.8%)	11 (31.4%)	
Child-Pugh grade			0.128
A	260 (91.2%)	29 (82.9%)	
B	25 (8.8%)	6 (17.1%)	
MELD			0.193
< 7.5	250 (87.7%)	28 (80.0%)	
≥ 7.5	35 (12.3%)	7 (20.0%)	

*Abbreviation*: *PHLF* post-hepatectomy liver failure, *BMI* body mass index, *PLT* platelet counts, *ALT* alanine transaminase, *AST* aspartate aminotransferase, *ALB* albumin, *PT* prothrombin time, *INR* international normalized ratio, *CREA* creatinine, *TBIL* total bilirubin, *ST* splenic thickness, *PVD* portal vein diameter, *PVT* portal vein thrombus, *EV* esophageal varices, *CSPH* clinically significant portal hypertension, *ICG R15* indocyanine green retention rate 15 min, *MELD* the model for end-stage liver disease

Data are expressed as means ± SD or *n* (%)

### Univariate and multivariate analyses of post-hepatectomy liver failure

To evaluate the independent predictive factors of PHLF during the perioperative period, we performed univariate and multivariate analyses to identify surgery-related and chronic liver disease-related variables (shown in Table 3). Univariate analysis showed that six variables were significantly associated with the development of PHLF, which included PLT, ST, TBIL, perioperative blood loss, major hepatic resection, and ICG R15. Multivariate logistic regression analysis indicated that ST, PLT, and major hepatic resection were identified as independent risk factors for predicting PHLF. It seems that ST (OR 3.39; 95% CI 1.374 to 8.362; *P* = 0.008) was a stronger independent risk predictor for PHLF than was ICG R15 (OR 2.130; 95% CI 0.893 to 5.081; *P* = 0.088).

**Table 3:** Univariable and multivariable analyses to identify predictors of PHLF in HBV-associated HCC patients

Variables	Univariate analysis		Multivariate analysis	
	Odds ratio	*P*	Odds ratio	*P*
Age, years	1.587 (0.749, 3.364)	0.228	1.372 (0.587, 3.206)	0.465
BMI, Kg/m2	0.886 (0.429, 1.831)	0.744	0.738 (0.323, 1.688)	0.472
Platelet count, × 10^9^/l	4.549 (2.205, 9.384)	< 0.001	2.608 (1.047, 6.497)	0.040
Operation time, min	0.878 (0.429, 1.796)	0.722	0.768 (0.339, 1.743)	0.529
ST, mm	5.465 (2.591, 11.529)	< 0.001	3.390 (1.374, 8.362)	0.008
Prothrombin time, s	1.408 (0.689, 2.879)	0.348	0.869 (0.382, 1.980)	0.739
TBIL, umol/l	2.981 (1.381, 6.435)	0.005	1.975 (0.816, 4.778)	0.131
Blood loss, ml	2.389 (1.081, 5.280)	0.031	1.655 (0.672, 4.074)	0.273
Major hepatic resection	2.263 (1.039, 4.928)	0.040	4.280 (1.624, 11.282)	0.003
ICG R15, %	4.119 (1.981, 8.561)	< 0.001	2.130 (0.893, 5.081)	0.088

*PHLF* post-hepatectomy liver failure, *HCC* hepatocellular carcinoma, *BMI* body mass index, *ST* splenic thickness, *TBIL* total bilirubin, *ICG R15* indocyanine green retention rate 15 min

Data in parentheses are 95% CI

### Discriminatory power of ST and ICG R15 for post-hepatectomy liver failure

**Fig. 1:**
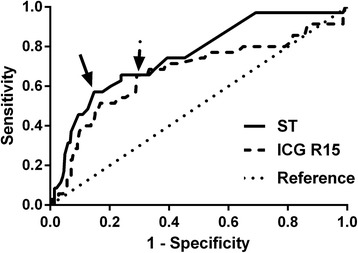
Receiver operating characteristic (ROC) curves of splenic thickness (ST) and indocyanine green retention rate 15 min (ICG R15) for predicting post-hepatectomy liver failure with HCC patients. Arrows show optimal cut-off values for ST 43.5 mm (sensitivity of 57.1%, specificity of 82.5%) and ICG R15 7.95(sensitivity of 65.7%, specificity of 70.9%). Area under the curve 0.754 for ST versus 0.670 for ICG R15

The area under the ROC curve of the ST (AUC 0.754; 95% CI 0.667 to 0.841; *P* < 0.001) for predicting PHLF was greater than that of the ICG R15 (AUC 0.670; 95% CI 0.560 to 0.779; *P* < 0.001) (shown in Fig. 1). The optimal cut-off value of ST was 43.5 mm, with a sensitivity of 57.1% and a specificity of 82.5% for predicting PHLF. Regarding the critical value of ST and ICG R15 (shown in Table 4), patients with a ST of 43.5 mm or above had a higher incidence of PHLF than patients with ST less than 43.5 mm, 20 (28.6%) of 70 patients and 15 (6%) of 250 patients, respectively, (OR 6.267; *P* < 0.001). Although there was a significant difference in the incidence of PHLF between patients with higher ICG R15 levels (≥ 7.95%) and those with lower ICG R15 levels (<  7.95%) (OR 4.665; *P* < 0.001), the results suggested that the predictive value of ST was better than that of ICG R15.

**Table 4:** The chi-square test in 2 × 2 table for the cut-off value of PHLF

Variables	PHLF (−) (*n* = 285)	PHLF (+) (*n* = 35)	Odds ratio	*P*
Splenic thickness			6.267	< 0.001
ST < 43.5 mm (*n*)	235 (82.5%)	15 (42.9%)		
ST ≥ 43.5 mm (*n*)	50 (17.5%)	20 (57.1%)		
ICG R15			4.665	< 0.001
ICG R15 < 7.95% (*n*)	202 (70.9%)	12 (34.3%)		
ICG R15 ≥ 7.95% (*n*)	83 (29.1%)	23 (65.7%)		

*PHLF* post-hepatectomy liver failure, *ST* splenic thickness, *ICG R15* indocyanine green retention rate 15 min

### Correlation between ST and other preoperative variables

**Fig. 2:**
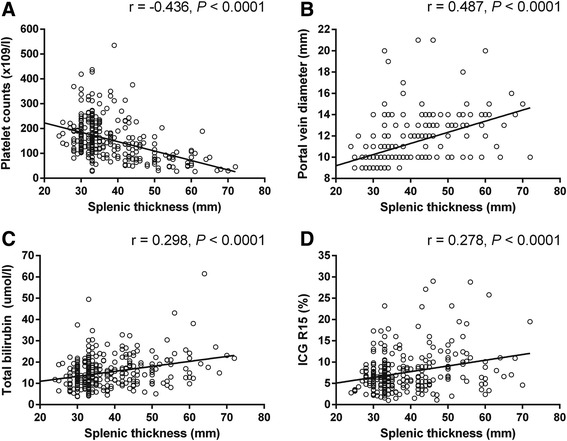
Scatterplots show correlations between splenic thickness (ST) and **a** platelet counts (PLT), **b** portal vein diameter (PVD), **c** total bilirubin (TBIL), and **d** ICG R15

Spearman’s rank correlation analysis was used to analyze the correlation between ST and PLT, TBIL, portal vein diameter (PVD), and ICG R15. ST was positively correlated with TBIL, ICG R15, and PVD and negatively correlated with PLT. As shown in Fig. 2, although correlations with these four variables achieved statistical significance, ST was strongly correlated with preoperative PLT (*r* − 0.433; *P* < 0.001) and PVD (*r* 0.487; *P* < 0.001) and weakly correlated with ICG R15 (*r* 0.202; *P* < 0.001).

### Postoperative morbidity and mortality

To further assess the independent predictive risk value of ST in the early postoperative period, we performed multivariate logistic regression analyses to recognize clinicopathologic variables associated with perioperative morbidity and mortality (Table 5). ST was associated with an almost sixfold increased risk for developing perioperative complications after hepatectomy (OR 5.678; 95% CI 2.873 to11.224; *P* < 0.001). Of the entire cohort, seven patients (2.19%) died within 30 days of hepatectomy. Four patients died because of PHLF followed by multiple organ failure and sepsis, and three patients died before POD 3 because of multiple organ failure following massive hemorrhage. Mortality within 30 days after liver resection was almost 13-fold higher in patients with a thickened spleen (OR 13.007; 95% CI 1.238 to 136.627; *P* = 0.033). In addition, the results suggested that ICG R15 was statistically insignificant in predicting both morbidity and mortality.

**Table 5:** Possible risk factors associated with postoperative morbidity and mortality

Variables	Morbidity			Mortality		
	Odds ratio	95% CI	*P*	Odds ratio	95% CI	*P*
Platelet count, × 10^9^/l	3.691	1.739–7.832	0.001	1.004	0.127–7.947	0.997
Tumor size, cm	1.639	0.810–3.319	0.170	0.053	0.004–0.699	0.026
Splenic thickness, mm	5.678	2.873–11.224	< 0.001	13.007	1.238–136.627	0.033
Major hepatic resection	1.217	0.528–2.808	0.645	17.806	2.043–155.189	0.009
ICG R15, %	1.437	0.750–2.746	0.272	2.448	0.375–15.975	0.350

*ICG R15* indocyanine green retention rate 15 min

## Discussion

Given that there is a high mortality associated with PHLF, there is an increased interest in identifying HCC patients who are at risk for hepatic dysfunction or failure at the preoperative stage. An effective tool to achieve this target is volumetric analysis using CT scans. Nevertheless, PHLF remains a severe complication of hepatic resection, which occurs in approximately 8% of the patients undergoing major hepatectomy. Thus, early recognition and therapy are crucial to enhance the survival of patients in the setting of PHLF. The observation of preoperative splenomegaly and hypersplenism in patients with liver cirrhosis or chronic hepatitis prompted an investigation into whether splenic thickness is a predictor of hepatic resection. In the present study, we demonstrated for the first time that ST was associated with morbidity, PHLF, and mortality after hepatectomy, particularly in patients with ST ≥ 43.5 mm, where hepatic resection resulted in a significantly higher proportion of PHLF. Meanwhile, ST may be superior to the ICG R15 level in predicting the development of PHLF in HBV-associated HCC patients.

The spleen, though as a secondary peripheral lymphoid organ in the human body, has been considered unnecessary for life so far. Nevertheless, the spleen serves extremely significant immunological and hematological functions and is closely related to the liver [21]. Owing to the spleen is anatomically linked to the liver via the portal vein system; once the histology and hemodynamics of hepatic had changed, so did the spleen. Many previous studies have introduced spleen size as a diagnostic criterion for cirrhosis [22].The former finding of Chen X et al. [23] in a cohort of hepatitis B cirrhosis patients showed the spleen volume and spleen multidimensional index increased with increasing Child-Pugh class of cirrhosis. Tsushima et al. [24] reported that the spleen longitudinal diameter in patients with a non-alcoholic fatty liver disease was significantly higher than in healthy subjects. In addition, Murata Y et al. [25] showed that patients with primary biliary cirrhosis (PBC) tended to have a larger spleen, especially in PBC patients who developed symptoms. However, some reports indicated that the spleen size in patients with alcoholic cirrhosis was smaller than in those with hepatitis C and non-alcoholic steatohepatitis cirrhosis [22, 26].

Child-Pugh grade and MELD scores have been widely used to predict the risk of death and complications in patients with liver diseases, sometimes been considered as a further aspect of the liver cirrhosis severity [27]. In our results, the patients with thickened spleen have a higher proportion of CP-B than normal group (15.5 vs. 7.2%, respectively), so does MELD (22.7 vs. 9.0%, respectively). And with increasing thickness of the spleen, the patients get higher MELD scores. This was in accordance with the recent study reported by Haliloglu N [28]. Meanwhile, a thicker spleen seems to more likely to be in the context of cirrhosis in our present study. It can be said that the thicker the spleen is the more possibility to give results by abnormal liver in histology and function.

Among the various qualitative tests for liver function, ICG R15 has long been considered as one of the most powerful predictors of post-hepatectomy mortality [29]. However, ICG R15 may be an inaccurate predictor under certain conditions, such as in patients with jaundice or heart failure. The value of splenic thickness in predicting PHLF was comparable to that of the ICG R15 level. According to the ROC curve analysis for predicting PHLF, the AUC for ST and ICG R15 level was 0.754 and 0.670, respectively, which suggested that ST is a superior predictor of PHLF compared with the ICG R15 level. Although the sensitivity of ST was 57.1% and relatively lower than the 65.7% for the sensitivity of the ICG R15 level, a higher specificity of ST may help the surgeon develop a better treatment plan, resulting in patients receiving treatment who would otherwise not be considered suitable for surgery based on the assessment of ICG R15. Moreover, the present study showed that ST was associated with morbidity and mortality after liver resection in HCC patients, which was not the case with the ICG R15 level. Thus, it can be concluded that ST was a better predictor of PHLF and other post-hepatectomy outcomes than the ICG R15 level. Postoperative complications, such as bleeding, abdominal infection, ascites, and liver dysfunction, will inevitably prolong the hospital stay, increase the cost of treatment, and even lead to death. Therefore, surgical procedures involving liver resection in patients with ST ≥ 43.5 mm should be given critically important perioperative consideration.

In general, patients with a greater degree of cirrhosis have a higher portal venous pressure (PVP). Recently, CSPH, as a surrogate measure of PVP, has been demonstrated to have the ability to predict the incidence of PHLF [16]. Our study showed that CSPH is a risk factor for hepatectomy because the incidence of CSPH in patients with PHLF was 54.3% (19/35) versus 24.2% (69/285) in patients without PHLF. Indeed, 82.9% of the patients with PHLF had liver cirrhosis compared with 69.8% of the patients without PHLF. Furthermore, the incidence of CSPH was statistically significantly higher in the thickened spleen group and was accompanied by high TBIL and a greater presence of cirrhosis. In clinical practices, splenomegaly is usually defined as a longitudinal diameter > 12 cm [30]. Prassopoulos P and colleagues indicated that splenic thickness had a strong correlation with splenomegaly [31]. Furthermore, splenomegaly was viewed as a surrogate marker of portal hypertension in a previous study [32]. In our study, in addition to splenomegaly, the clinical manifestations of portal hypertension included thickening of the portal veins and faster blood flow [33]. A previous study suggested that portal vein diameter was an independent risk factor for CSPH and variceal bleeding [34]. We found a positive correlation between ST and PVD, which indicates that patients with greater ST may have higher portal pressure, although the latter was not determined in this study.

A negative correlation between ST and PLT was found in this study, which was evident from the finding that a low platelet count was associated with greater ST. This negative correlation in our study is in agreement with the thrombocytopenia and splenomegaly that arise due to the sequestration and destruction of platelets in liver cirrhosis [6]. Several clinical studies have indicated that preoperative thrombocytopenia is a risk factor associated with postoperative complications and mortality [35, 36]. This was consistent with our result that preoperative PLT was significantly lower in the PHLF group than in the non-PHLF group. Furthermore, hypersplenism leading to thrombocytopenia, which is frequent in patients with cirrhosis due to portal hypertension [37], may be a reason for the ability of ST to predict PHLF.

This study has certain limitations. First, we enrolled only HBV-infected HCC patients from China, and therefore, the study may suffer selection bias. Other causes of HCC include HCV infection and alcoholism, both of which are prevalent mainly in Europe and North America [38] and may change the prediction value of splenic thickness in PHLF. Second, parameters such as spleen length, spleen width, or spleen index, which are indicative of splenomegaly, were not measured by CT in this study. Finally, because this study was a single-study site retrospective analysis of clinical data, future research will require a multicenter validation of our findings.

## Conclusions

We demonstrated firstly that the thickness of the spleen is a strong predictor of the occurrence of PHLF in HBV-associated HCC patients undergoing hepatic resection. Furthermore, ST may be a better predictor of PHLF compared with ICG R15.

## Abbreviations

ALB: Albumin
ALT: Alanine transaminase
AST: Aspartate aminotransferase
AUC: Area under the receiver operating characteristic curve
BMI: Body mass index
CI: Confidence interval
CP: Child-Pugh
CREA: Creatinine
CSPH: Clinically significant portal hypertension
CT: Computed tomography
EV: Esophageal varices
HBsAg: Hepatitis B surface antigen
HBV: Hepatitis B virus
HCC: Hepatocellular carcinoma
HCV: Hepatitis C virus
ICG R15: Indocyanine green retention rate 15 min
INR: International normalized ratio
MELD: Model for end-stage liver disease
MRI: Magnetic resonance imaging
OR: Odds ratio
PBC: Primary biliary cirrhosis
PHLF: Post-hepatectomy liver failure
PLT: Platelet count
PT: Prothrombin time
PVD: Portal vein diameter
PVP: Portal venous pressure
PVT: Portal vein thrombus
ROC: Receiver operating characteristic
SD: Standard deviation
ST: Splenic thickness
TBIL: Total bilirubin
